# Associations between serum homocysteine levels and anxiety and depression among children and adolescents in Taiwan

**DOI:** 10.1038/s41598-017-08568-9

**Published:** 2017-08-21

**Authors:** Kuo-Hsuan Chung, Hung-Yi Chiou, Yi-Hua Chen

**Affiliations:** 1 Department of Psychiatry, Taipei Medical University Hospital, 252 Wu-Hsing St., Taipei, 110 Taiwan; 2 Psychiatric Research Center, Taipei Medical University Hospital, 252 Wu-Hsing St, Taipei, 110 Taiwan; 3 Department of Psychiatry, School of Medicine, College of Medicine, Taipei Medical University, 250 Wu-Hsing St, Taipei, 110 Taiwan; 4 School of Public Health, College of Public Health, Taipei Medical University, 250 Wu-Hsing St, Taipei, 110 Taiwan

## Abstract

Although evidence suggests that homocysteine levels are elevated in severe mental illness in children, findings regarding homocysteine levels in youth with anxiety and depression are scarce. Therefore, this study examined the association of homocysteine levels with anxiety and depression in a community sample of students aged 6–13 years. In total, 649 students were selected from the first, fourth, and seventh grades of schools in Taipei, Taiwan, in 2010. These students completed a hospital-based health examination, which included physical examination, blood sample collection, and questionnaire administration. The data were analysed through multiple linear regression. Among the seventh-grade boys, both depression (adjusted β = 0.044, 95% confidence interval (CI) = 0.004–0.084) and anxiety (adjusted β = 0.052, 95% CI = 0.013–0.091) were independently associated with increased homocysteine levels. In further dichotomisation, compared with students with low anxiety levels, those with moderate to high anxiety levels were significantly positively associated with elevated serum homocysteine levels (adjusted β = 0.091, 95% CI = 0.003–0.180). Our results suggest that increased depression and anxiety may be positively associated with higher serum homocysteine levels in older boys. Our results provide essential data on the biological aspects underlying anxiety and depression in the studied population.

## Introduction

Epidemiologic studies focusing on mental illness in children and adolescents have indicated a high prevalence of anxiety and depression, which occur together (i.e., as a comorbidity)^1^ in up to 3.4% of cases of anxiety and 9.5% of cases of depression, leading to functional impairment and negative consequences^2^. However, fewer than half of these patients with mental disorders receive speciality management and intervention because of several obstacles to the provision of adequate mental health treatment^3^. In addition to some psychosocial barriers, such as distance, financial barriers, sociocultural barriers, language- and ethnicity-related barriers, and a lack of knowledge and awareness^4^, inadequate research on the biological mechanisms underlying anxiety and depression in youth may contribute to this predicament.

Homocysteine, a sulphurated amino acid derived from ingested methionine, is a component of the homocysteine–methionine cycle, which mediates methylation and plays a crucial role in maintaining the biochemical balance within the central nervous system^5^. A growing body of research has demonstrated that elevated homocysteine levels may be associated with an increased risk of cardiovascular, neurological, and psychiatric diseases including coronary artery disease, stroke, dementia, and depression in adults^5–8^. Several studies from Western countries have demonstrated an increase in homocysteine levels in adults and elderly patients with depression^9–11^; however, no consensus exists on a similar association in such adults in Eastern countries^12^. Despite evidence showing elevated levels of homocysteine in severe mental illness in children and adolescents^9, 13–15^, findings concerning homocysteine in anxiety and depression in children and adolescents are scant^15^.

Therefore, this study was conducted to examine the association of homocysteine levels in youth with anxiety and depression in the metropolitan Taipei area of Taiwan. We hypothesised that higher anxiety and depression may be associated with elevated serum homocysteine levels.

## Methods

### Participants

This cross-sectional study involved students from eight elementary schools and two junior high schools in metropolitan Taipei, Taiwan. We recruited students in the first, fourth, and seventh grades because first graders (aged 6–7 years) and seventh graders (aged 12–13 years) in Taiwan are at a crucial life stage, as they start attending elementary schools and junior high schools, respectively; the fourth graders are at the midpoint and thus enable an examination of changes in the other two groups. Of the 3,500 students attending the first and fourth grade in the eight elementary schools, 10% were randomly invited to participate in a health examination (which included physical examination, blood sample collection, and questionnaire administration) at Taipei Medical University Hospital. The same procedure was applied for adolescents attending the seventh grade in the two junior high schools. Of these students, those who were willing to participate in the study were recruited. In total, 149, 160, and 340 students in the first (aged 6–7 years), fourth (aged 9–10 years), and seventh (aged 12–13 years) grades, respectively, in the academic year 2010 completed the health examination and were included in our analysis.

This study is a part of an integrated project on child weight, which was approved by the Ethics Committee of Taipei Medical University Hospital, and the study was conducted in accordance with the approved guidelines. Written informed consent was obtained from each student’s parents before inclusion in the study. The children’s informed consent for participation was also acquired.

### Measurement of physiological characteristics

The physical examination and anthropometric measurements were conducted in the hospital. A combined wall-mounted stadiometer and metric balance scale (NAGATA K-100/P-100) was used to assess the participants’ body height and weight without shoes to the nearest 0.5 cm and 0.1 kg, respectively. Body mass index (BMI) was calculated as body weight (kg) divided by height squared (m^2^). After the participants had been seated quietly and rested for 5 min, trained personnel measured the systolic blood pressure (SBP) and diastolic blood pressure (DBP) of each student in duplicate on the right arm by using an automated sphygmomanometer (Terumo ES-P2000).

Following a 10–12-h fasting period, peripheral blood specimens (5–8 mL) were collected from these participants by using a vacuum syringe. Samples were separated into red blood cells and serum and were frozen at −80 °C for measuring lipid levels, lipoprotein content, serum liver enzymes, and fasting blood glucose. Serum homocysteine was measured using an enzymatic assay presented by Chan *et al*.^16^. Specifically, the assay used a crude lysate of *Escherichia coli* containing the recombinant enzyme methionine c-lyase, a commercially available chromophore. For the assay, 96-well microtiter plates were used either manually or with an automated Tecan analyser. Within-individual and between-individual precision levels were <10% for the coefficient of variation. Correlations between the results obtained using the enzymatic method and those obtained using a reference high-performance liquid chromatography procedure were found to be appropriate (r > 0.9)^16^. Furthermore, serum levels of total cholesterol (T-Chol) and triglycerides (TGs) were measured using an autoanalyser (Hitachi 737, USA) with reagents acquired from Boehringer Mannheim Diagnostics (Indianapolis, IN, USA). Values of high- and low-density lipoprotein (HDL and LDL, respectively) cholesterol were also assessed. Other blood tests of the metabolic profile (glucose and insulin levels), liver function markers (aspartate transaminase (AST) and alanine aminotransferase (ALT)), and renal function markers (creatinine and urea N) were performed in clinical laboratories. An electrocardiogram (ECG) machine was used and read by specialists, and the results were recoded as normal, arrhythmia, and abnormal findings other than arrhythmia.

### Measurement of psychological and other characteristics

We used the Beck Youth Inventories, second edition (BYI-II)^17, 18^, to assess the children’s self-reported thoughts, feelings, and behaviours related to emotional dysfunction. Two subscales, with 20 questions each, were used to measure the children’s experiences in the psychological domains of depression (assessing negative thoughts regarding the self, life, and the future and feelings of sadness and guilt) and anxiety (assessing worries regarding school performance, the future, negative reactions of others, and fears). A 4-point Likert scale was used for each item, with responses ranging from ‘never’ (0) to ‘always’ (3). Total scores were summed for each subscale, with higher scores indicating a higher tendency of a particular psychological domain. Furthermore, the raw scores were transformed (standardised) into T scores (with a mean of 50 and standard deviation of 10) to allow profiling across scales and comparison of one’s score with other children of the same age and sex. Both reliability (internal consistency and test–retest reliability) and validity were satisfactory for all age groups on both subscales^18^ and the overall scale of the Chinese version of the BYI-II^19^. Finally, a face-to-face interview was conducted by well-trained interviewers to collect information on sociodemographics and the children’s lifestyle factors, including the frequency of fruit and vegetable consumption (times/week).

### Data analysis

Summary statistics were examined for demographic characteristics and serum biochemistry values and stratified by the children’s sex and grade. The Shapiro–Wilk test was used to test data normality. Independent-sample t tests and one-way analysis of variance (with Scheffe’s post-hoc tests) were used for variables that exhibited normal distributions (i.e., depression and anxiety scores, SBP, DBP, total cholesterol, HDL, LDL, urea N, and BMI), whereas variables that did not exhibit normal distributions (i.e., homocysteine, AST, ALT, triglyceride, fasting blood glucose, and creatinine levels) were analysed using the Mann–Whitney U test and the Kruskal–Wallis test. Pearson’s χ^2^ test of significance was used to examine differences in child traits on the basis of the children’s sex and grade. In addition to integrating the findings of previous reports, all variables with potential risk associations (P < 0.2) were included in the subsequent multiple linear regression analyses to assess the association of homocysteine levels with the children’s depression and anxiety. Variables to be included in the final models were selected on the basis of model fittings. Homocysteine level readings were natural log-transformed to obtain a normal distribution before analyses. To highlight the differences, the results were calculated for 10-unit (1 SD) increments of depression and anxiety scores. Because the interaction terms of sex and grade with the main independent variables (i.e., depression and anxiety) were statistically significant, separate multiple linear regression analyses stratified by children’s sex and grade were performed.

Finally, in addition to the continuous depression and anxiety T scores, as generally preferred in the statistical modelling with the provision of more variability, we attempted to detect children with psychological difficulties above the ‘warning line’ (i.e., high-risk groups), who deserve additional attention. To this end, we used cutoff points suggested in the BYI-II manual to categorise the children into two groups: those with moderate to high levels of depression or anxiety (i.e., the high-risk group) and those with low levels of depression or anxiety^15, 16^. Because depression and anxiety are highly comorbid, we further classified the children into four groups—those with both depression and anxiety, with depression only, with anxiety only, and with neither (hereafter referred to as ‘none’)—to assess the effects of depression and anxiety simultaneously. The regression coefficients (β) and 95% confidence intervals (CIs) are reported. All tests of significance were two-tailed, with P < 0.05 being considered significant. Data were analysed using Statistical Package for the Social Sciences (SPSS v19; SPSS, Chicago, IL, USA).

### Ethical Approval

This study is a part of an integrated project on child obesity, which was approved by the Ethics Committee of Taipei Medical University Hospital. Written informed consent was obtained from each student’s parents before inclusion in the study. The children’s informed consent for participation was also acquired.

### Data Availability Statement

A request for access to the study data can be made by contacting the corresponding author. Access can be granted subject to the Institutional Review Board (IRB) and the research collaborative agreement guidelines. This is a requirement mandated for this research study by our ethnics committee and funders.

## Results

Of the participants in our study, 149, 160, and 340 students were in the first, fourth, and seventh grades, respectively, representing a total of 346 (53.3%) boys and 303 (46.7%) girls. Table 1 lists the distributions of the characteristics and serum biochemical values of all students and students stratified by sex. Boys were more likely to have higher levels of homocysteine, AST, ALT, HDL, urea N, and BMI and lower values of triglycerides (all P < 0.05) than were girls.

**Table 1:** Sex-wise distributions of characteristics and serum biochemical values.

	**Total (n = 649)**		**Boys (n = 346)**		**Girls (n = 303)**		**P**
	**Range**	**Median**	**Range**	**Median**	**Range**	**Median**	
Homocysteine (mol/L)	1.87–27.9	8.61	1.87–27.9	8.86	4.73–22.99	8.38	0.012^a,*^
AST (IU/L)	11–124	21	11–124	22	11–54	20	<0.001^a,***^
ALT (IU/L)	4–271	13	4–271	14	5–106	12	<0.001^a,***^
TGs (mg/dL)	25–356	64	25–263	62	29–356	66	0.044^a,*^
Fasting blood glucose (mg/dL)	57–363	89	57–143	90	69–363	89	0.051^a^
Creatinine (mg/dL)	0.3–0.9	0.5	0.3–0.9	0.5	0.3–0.8	0.5	0.037^a,*^
	**Mean**	**SD**	**Mean**	**SD**	**Mean**	**SD**	**P**
Depression	46.75	9.77	46.87	10.05	46.62	9.46	0.746^b^
Anxiety	48.67	9.68	48.54	9.86	48.82	9.48	0.711^b^
SBP (mmHg)	108.67	10.87	109.26	10.55	108.00	11.21	0.143^b^
DBP (mmHg)	62.71	9.06	62.55	8.83	62.90	9.33	0.626^b^
T-Chol (mg/dL)	166.56	29.85	166.38	31.57	166.76	27.82	0.875^b^
HDL (mg/dL)	57.44	12.56	58.42	12.58	56.31	12.47	0.034^b*^
LDL (mg/dL)	94.88	25.03	94.16	26.32	95.71	23.49	0.435^b^
Urea N (mg/dL)	11.80	2.71	12.12	2.84	11.43	2.52	0.001^b,**^
BMI (kg/m^2^)	20.30	4.21	20.64	4.30	19.91	4.08	0.027^b,*^
	**n** ^d^	**%**	**n** ^d^	**%**	**n** ^d^	**%**	**P**
ECG							0.193^c^
Normal	499	79.6	260	77.8	239	81.6	
Arrhythmia	30	4.8	14	4.2	16	5.5	
Other abnormality	98	15.6	60	18.0	38	13.0	
Grade							0.776^c^
First	149	23.0	76	22.0	73	24.1	
Fourth	160	24.7	88	25.4	72	23.8	
Seventh	340	52.4	182	52.6	158	52.1	
Fruit frequency (times/week)							0.434^c^
0–5	148	23.4	84	24.9	64	21.7	
6–10	235	37.2	130	38.6	105	35.6	
11–15	139	22.0	70	20.8	69	23.4	
>16	110	17.4	53	15.7	57	19.3	
Vegetable frequency (times/week)							0.564^c^
0–5	78	12.3	43	12.7	35	11.8	
6–10	142	22.3	72	21.2	70	23.6	
11–15	214	33.6	109	32.2	105	35.4	
>16	202	31.8	115	33.9	87	29.3	

*P < 0.05; **P < 0.01; ***P < 0.001.

^a^Results from a Mann–Whitney U test.

^b^Results from a t test.

^c^Results from a chi-square test.

^d^Total counts may vary because of missing data.

AST, aspartate transaminase; ALT, alanine aminotransferase; TGs, triglycerides; SBP, systolic blood pressure; DBP, diastolic blood pressure; T-Chol, total cholesterol; HDL, high-density lipoprotein; LDL, low-density lipoprotein; ECG, electrocardiogram.

Table 2 presents a comparison of the serum biochemical values and some sociodemographic characteristics of the children by grade; significant differences were observed in homocysteine, AST, ALT, fasting blood glucose, and creatinine levels (all P < 0.001). The seventh graders were significantly more likely to have higher levels of depression, anxiety, SBP, DBP, and BMI and to have lower levels of total cholesterol, HDL, LDL, and urea N (all P < 0.01), compared with the other two groups. Furthermore, significantly different distributions of ECG and fruit consumption frequency were observed among the three groups (all P < 0.05).

**Table 2:** Grade-wise distributions of characteristics and serum biochemical values.

	First grade (n = 149)		Fourth grade (n = 160)		Seventh grade (n = 340)		P
	Range	Median	Range	Median	Range	Median	
Homocysteine (mol/L)	5.16–13.09	7.99	1.87–22.99	8.02	3.2–27.9	9.31	<0.001^a***^
AST (IU/L)	15–75	25.00	14–124	23.00	11–95	20.00	<0.001^a***^
ALT (IU/L)	5–82	14.00	7–271	15.00	4–178	12.00	<0.001^a***^
TGs (mg/dL)	26–263	58.00	25–356	66.00	26–353	65.00	0.144^a^
Fasting blood glucose (mg/dL)	69–173	85.00	75–363	89.00	57–140	91.00	<0.001^a***^
Creatinine (mg/dL)	0.3–0.7	0.50	0.4–0.7	0.50	0.4–0.9	0.60	<0.001^a***^
	**Mean**	**SD**	**Mean**	**SD**	**Mean**	**SD**	**P**
Depression	44.96	8.50	45.99	9.73	47.89	10.18	0.005^b**^ 7 > 1
Anxiety	47.30	8.56	46.74	8.36	50.17	10.46	<0.001^b***^ 7 > 1,4
SBP (mmHg)	101.73	10.33	108.99	12.09	111.45	9.10	<0.001^b***^ 7 > 4 > 1
DBP (mmHg)	60.12	9.21	63.36	10.11	63.50	8.30	0.001^b**^ 4,7 > 1
T-Chol (mg/dL)	172.91	29.70	175.00	30.94	159.97	27.86	<0.001^b***^ 1,4 > 7
HDL (mg/dL)	60.45	11.43	58.45	12.79	55.69	12.65	<0.001^b***^ 1 > 7
LDL (mg/dL)	100.04	26.14	100.21	24.69	90.24	23.83	<0.001^b***^ 1,4 > 7
Urea N (mg/dL)	12.36	2.65	12.00	2.92	11.47	2.60	0.002^b**^ 1 > 7
BMI (kg/m^2^)	19.05	4.02	20.98	4.59	20.52	4.00	<0.001^b***^ 4,7 > 1
	n^d^	%	n^d^	%	n^d^	%	P
ECG							<0.001^c***^
Normal	80	58.4	105	70	314	92.4	
Arrhythmia	15	10.9	7	4.7	8	2.4	
Other abnormality	42	30.7	38	25.3	18	5.3	
Sex (male)	76	51.0	88	55.0	182	53.5	0.776^c^
Fruit frequency (times/week)							0.020^c*^
0–5	30	21.3	27	17.8	91	26.8	
6–10	55	39.0	66	43.4	114	33.6	
11–15	37	26.2	24	15.8	78	23.0	
>16	19	13.5	35	23.0	56	16.5	
Vegetable frequency (times/week)							0.110^c^
0–5	17	11.8	11	7.2	50	14.7	
6–10	30	20.8	36	23.5	76	22.4	
11–15	56	38.9	59	38.6	99	29.2	
>16	41	28.5	47	30.7	114	33.6	

*P < 0.05; **P < 0.01; ***P < 0.001.

^a^Results from a Kruskal–Wallis test.

^b^Results from a one-way analysis of variance test.

^c^Results from a chi-square test.

^d^Total counts may vary because of missing data.

AST, aspartate transaminase; ALT, alanine aminotransferase; TGs, triglycerides; SBP, systolic blood pressure; DBP, diastolic blood pressure; T-Chol, total cholesterol; HDL, high-density lipoprotein; LDL, low-density lipoprotein; ECG, electrocardiogram.

Because the interaction terms of sex and grade with depression and anxiety were statistically significant, multiple linear regression models were fitted and examined by sex and grade (Table 3). In the models without adjustment, depression was associated with an increase in homocysteine levels among seventh-grade boys, and anxiety was associated with an increase in homocysteine levels among fourth-grade girls and seventh-grade boys. The association remained for the seventh-grade boys after the models were adjusted for HDL, AST, BMI, SBP, fasting blood glucose, ECG status, fruit consumption frequency, and vegetable consumption frequency. Specifically, among the seventh-grade boys, a 10-unit (1 SD) increase in the depression and anxiety scores was independently associated with a mean increase of 0.044 (95% CI = 0.004–0.084) and 0.052 (95% CI = 0.013–0.091) homocysteine units (in log scale), respectively.

**Table 3:** Effects of d epression and anxiety scores on homocysteine by sex and grade in Taiwan: results from linear regression models.

	First-grade boys (n = 76)	First-grade girls (n = 73)	Fourth-grade boys (n = 88)	Fourth-grade girls (n = 72)	Seventh-grade boys (n = 182)	Seventh-grade girls (n = 158)
	β (95% CI)	β (95% CI)	β (95% CI)	β (95% CI)	β (95% CI)	β (95% CI)
*Model 1*						
Depression	−0.029 (−0.091 to 0.033)	0.029 (−0.027 to 0.086)	0.030 (−0.037 to 0.097)	0.042 (−0.041 to 0.124)	0.045* (0.006 to 0.084)	−0.017 (−0.055 to 0.022)
Anxiety	−0.023 (−0.079 to 0.034)	0.015 (−0.050 to 0.079)	0.030 (−0.050 to 0.110)	0.095* (0.001 to 0.189)	0.049* (0.010 to 0.087)	0.006 (−0.031 to 0.043)
*Model 2*						
Depression	0.005 (−0.071 to 0.082)	0.019 (−0.052 to 0.090)	0.062 (−0.019 to 0.143)	0.022 (−0.076 to 0.120)	0.044* (0.004 to 0.084)	−0.018 (−0.058 to 0.023)
Anxiety	0.004 (−0.072 to 0.080)	0.005 (−0.076 to 0.086)	0.034 (−0.066 to 0.134)	0.070 (−0.041 to 0.182)	0.052** (0.013 to 0.091)	0.006 (−0.032 to 0.044)

Model 1: Crude model. Results were calculated per 10-unit (1 SD) increment of depression and anxiety (continuous) scores.

Model 2: For both models of depression and anxiety, results were obtained after adjustment for HDL, AST, BMI, ECG, systolic blood pressure, fasting blood glucose, fruit consumption frequency, and vegetable consumption frequency. Results were calculated corresponding to 10-unit (1 SD) increments in depression and anxiety scores.

*P < 0.05; **P < 0.01; ***P < 0.001.

CI: confidence interval.

In addition, to detect children with high levels of psychological difficulties (i.e., the high-risk groups), depression and anxiety were further dichotomised into ‘moderate to high’ and ‘low’. In the regression models, the only significant association was among adolescent boys in the seventh grade. Compared with the low anxiety level, the moderate to high anxiety level was significantly positively associated with higher homocysteine levels (adjusted β = 0.091, 95% CI = 0.003–0.18; data not shown in table). Finally, because depression and anxiety are highly comorbid, we classified the children into four groups—both depression and anxiety, depression only, anxiety only, and none—to analyse these two traits simultaneously. Although no group differed significantly in the adjusted regression models, a marginal significance was observed among seventh-grade boys. Specifically, compared with the none group, the both depression and anxiety group was positively associated with higher homocysteine levels (β = 0.11, 95% CI = −0.008–0.228, P = 0.06) among seventh-grade boys (data not shown in table).

## Discussion

According to our review of the literature, this is the first study to investigate the association of serum homocysteine levels with anxiety and depression among Asian children and adolescents. The major finding was that in seventh-grade boys (aged 12–13 years), both increased depression and anxiety were independently associated with an increase in homocysteine levels after adjustment for HDL, AST, BMI, SBP, fasting blood glucose, ECG status, fruit consumption frequency, and vegetable consumption frequency. We further examined the effects of a high-risk group that was likely to experience psychological difficulties on serum homocysteine. Regarding anxiety (but not depression), only older boys in the high-risk group tended to have higher serum homocysteine levels than their counterparts.

These findings are consistent with the findings of studies in Western countries, which have indicated that a significant interaction exists between homocysteine levels and age and sex in children and adolescents, with a divergence in homocysteine levels at approximately 10 years of age^20, 21^. Therefore, race and ethnicity do not appear to be related to serum homocysteine levels in children and adolescents^20^. A potential explanation for the specificity of our findings to boys is partially related to the biological interaction between sex hormones and homocysteine. Increased homocysteine levels were previously associated with increased androgen levels in boys^22^, whereas oestrogen levels were negatively correlated with homocysteine levels in women^23^. Thus, sex hormones may exert a similar effect on the association between depression and homocysteine.

Current research on the relationship between anxiety and homocysteine is scant. Although anxiety–depression comorbidity is common^1^, we demonstrated that anxiety may differ biologically from depression in terms of homocysteine levels in children and adolescents. The positive association of moderate to high anxiety levels with elevated homocysteine levels in older boys observed in our study is consistent with the findings of research conducted in adults. Levine *et al*.^24^ observed that the duration of posttraumatic stress disorder predicts serum homocysteine levels, and elevated levels of homocysteine in male patients with posttraumatic stress disorder may be related to pathophysiological aspects associated with the chronicity of this disorder. Similarly, Türksoy *et al*.^25^ reported that patients with obsessive–compulsive disorder might have higher homocysteine levels; however, the patient and control groups predominantly comprised female patients (88.6% and 86.4%, respectively). One possible mechanism underlying the link between homocysteine and anxiety may be related to brain oxidative status. A recent animal study indicated that hyperhomocysteinemia induced by methionine nutritional overload increases anxiety-related behaviour in rats, and the proanxiogenic effects could have resulted from oxidative stress in the rat brain^26^. Despite evidence that oxidative stress is associated with the dysregulation of homocysteine-related methylation reactions^5^, the causal relationship as well as the sex- and age-related differences between homocysteine and anxiety remain to be elucidated in children and adolescents.

Although we found the association between high depression and elevated homocysteine levels in boys, we could not observe the effect of a high-risk depression group on serum homocysteine among children and adolescents, in both boys and girls. Several studies have indicated hyperhomocysteinemia in patients with depression, particularly in adults and elderly individuals^9–11^. In a recent large population-based study examining 3544 women and 3189 men aged 35–66 years in Switzerland, elevated homocysteine levels were associated with lifetime major depressive disorder, particularly with remitted depression in men, but not in women^10^. Moreover, homocysteine levels may indicate a significant prevalence of hyperhomocysteinemia in bipolar depressed patients during an acute episode^11^, or homocysteine could be a biomarker for depression, particularly in the acute phase of acute coronary syndrome^27^. Concerning the effect of age, evidence from nationally representative data on US adults aged 20–85 years derived from the National Health and Nutrition Examination Survey for the period 2005–2006 indicates that overall, elevated depressive symptoms were not significantly related to homocysteine levels; however, in adults older than 50 years of both sexes, homocysteine levels were positively associated with a higher number of depressive symptoms^9^. This suggests that a stronger association may exist between homocysteine levels and depression in older populations, and a weaker or nonsignificant association may exist in younger populations^28^ and in children and adolescents. However, a cross-sectional study in China did not find this association in older adults^12^. Hence, continued research is essential to determine the effects of differences in race or ethnicity, age, and sex on the association between homocysteine levels and depression.

This study has several methodological limitations. First, because the study involved a cross-sectional design without a longitudinal follow-up, clarifying causal relationships between homocysteine levels and anxiety and depression was difficult. Second, our sample was a small subgroup of a much larger population; therefore, the results of this study should be applied cautiously to the general population. In addition, the sample size of the oldest group was nearly twice that of the younger group; therefore, the null findings in the younger group might be due to inadequate power. Nevertheless, the statistical power for our sample groups and sex and grade subgroups ranged from 0.7–0.9, which should be adequate for association detection. Studies with more balanced subgroups would help further clarify the differences in the relationship between emotional disturbances and serum homocysteine by age and sex.

Third, because the effects of depression and anxiety on homocysteine were examined separately by sex and grade in the linear regression models, the use of multiple statistical tests increases the likelihood of incorrectly rejecting a null hypothesis (i.e., an increase in the probability of a Type I error). After the application of the Bonferroni correction, only the adjusted effects of anxiety on homocysteine levels among seventh-grade boys sustained (P = 0.008 < 0.0083; i.e., 0.05/6). Therefore, our results, especially those pertaining to the effects of depression, should be conservatively interpreted. Additional studies are necessary to replicate our findings before their clinical application. Fourth, we used self-reported measurement in this study, which may have led to biases caused by the tendency of people to provide socially desirable responses and by differences in participants’ interpretations of anxiety and depression. A clinician rating or multi-informant approach should be considered in the future to enhance the validity and accuracy of the assessment. Nevertheless, both reliability (internal consistency and test–retest reliability) and validity were acceptable for all age groups on both the anxiety and depression scales as well as for the overall scale of the Chinese version of the BYI-II^18, 19^. Finally, data on the medication use of the participants were unavailable. Furthermore, the literature indicates that homocysteine levels are an independent risk factor for cardiovascular disease^7, 8^. Although we did not undertake an advanced survey of cardiovascular diseases in our sample, the prevalence of cardiovascular disease in children and adolescents is known to be relatively low. We adjusted our data for the ECG status as well as other potential confounding factors such as fruit and vegetable consumption frequency, serum lipid levels, and BMI.

In conclusion, in this Taiwanese investigation, we demonstrated that regarding anxiety, only older boys with moderate to high anxiety levels may have higher serum homocysteine levels than do boys with low anxiety levels. No association was observed between serum homocysteine levels and depression among both boys and girls. Sex and age differences should be considered in studies on homocysteine levels in children and adolescents. The results of our study enrich the limited data on biological aspects underlying anxiety and depression in this population. Further research is required to replicate our findings in other races and ethnicities and to clarify the role of homocysteine in anxiety and depression among children and adolescents.
